# Perceptions of stigma of pregnant individuals experiencing substance use disorder receiving prenatal care at magdalene clinic: a cross-sectional study

**DOI:** 10.1186/s12954-025-01377-8

**Published:** 2026-01-14

**Authors:** Hannah F. McKinnon, Melissa L. Fair, Jody Teel, Courtney Lubaczewski, Alison Kimura, Kimbley Smith, Kacey Eichelberger

**Affiliations:** 1 Institute for the Advancement of Community Health, Furman University, Hipp Hall 013, 3300 Poinsett Highway, Greenville, SC 29613 USA; 2 The University of South Carolina School of Medicine Greenville, Greenville, SC USA; 3 Maternal-Fetal Medicine Greenville, Prisma Health Upstate, Greenville, SC USA; 4 Serenity Village, The Phoenix Center, Greenville, SC USA; 5 Department of Obstetrics and Gynecology, Prisma Health Upstate, Greenville, SC USA

**Keywords:** Substance use disorder, Pregnancy, Stigma, Collaborative care, Trauma informed care, Prenatal care

## Abstract

**Background:**

Substance use disorder (SUD) is highly stigmatized with pregnant individuals experiencing substance-related stigma at greater levels than the general population due to perceived deviance from societal norms. Pregnant individuals experiencing stigma may be more likely to delay or receive inadequate prenatal care. The purpose of this cross-sectional study was to understand self-reported enacted, anticipated, and internalized stigma of pregnant individuals experiencing SUD receiving prenatal care at Magdalene Clinic, a collaborative, trauma-informed OB-GYN clinic.

**Methods:**

The Substance Use Stigma Mechanism Scale was administered to 226 individuals to measure past, present, and anticipated future experiences of substance use related stigma from family, healthcare providers, and self. Participant demographics and SUD diagnosis were abstracted from the participant’s electronic medical record. One-way ANOVAs with Tukey’s post hoc tests were conducted in January 2025 using SAS version 9.4 to analyze the relationships between stigma and all study variables. Additionally, Welch’s ANOVAs with Games-Howell post hoc tests were run where Levene’s tests indicated a variance of homogeneity.

**Results:**

Significantly higher internalized stigma scores (*n* = 226) were associated with initiation of prenatal care in the second (M = 3.28, 95% CI [3.06, 3.50]) or third trimester (M = 3.73, 95% CI [3.32, 4.15]), compared to the first trimester. Individuals who used opioids/stimulants (M = 2.10, 95% CI [1.85, 2.34]) reported significantly higher stigma from healthcare providers than those using other substances. Individuals using stimulants (M = 2.77, 95% CI [2.51, 3.03]) reported higher levels of stigma from their family compared to those using other substances. Those with higher educational attainment reported significantly higher levels of stigma from healthcare providers than those with less than a high school diploma/GED (M = 1.64, 95% CI [1.42, 1.87]).

**Conclusions:**

These findings suggest that higher internalized and source-specific stigma, particularly for opioid and stimulant use, may contribute to delayed prenatal care and highlight the need for stigma-reducing interventions for pregnant individuals with SUD. Additionally, these findings suggest that pregnant individuals with social advantages like higher educational attainment may face unique substance-related stigma. These results inform the need for supportive, empathetic healthcare that is need specific and responsive to the diverse experiences of those experiencing pregnancy and SUD.

## Background

Stigma stems from deviance of what society considers normal and demoralizes an individual based on certain characteristics [1]. This definition of stigma by Goffman (1986) posits that there are different types of stigmas; the most salient being personal characteristics/traits viewed as undesirable or flawed, such as substance use disorder (SUD) or mental illness. SUD is a chronic medical condition involving repetitive, uncontrolled use of substances, leading to physiological impairment or distress [2]. Despite being a treatable chronic condition [2], SUD remains highly stigmatized with several studies indicating it is among one of the most stigmatized chronic medical conditions [3–6]. Stigma is especially high among pregnant individuals due to societal perceptions of SUD being a personal moral failure [6], assumptions of inadequate mothering [7], and gender expectations [8].

Pregnancy represents a particularly vulnerable time for persons with or at risk for SUD, and estimates are that approximately 5% of pregnant individuals within the United States (U.S.) use one or more substances during pregnancy [9, 10]. Substance use during pregnancy presents risks to both the baby (i.e., preterm birth, neonatal abstinence syndrome) and the pregnant individual [11, 12]. In fact, mental health and SUDs were responsible for 23% of U.S. maternal deaths in 2022, exceeding deaths from other pregnancy complications such as excessive bleeding or cardiovascular conditions [13]. Between 2017 and 2020, maternal deaths in the U.S. resulting from drug overdose increased by 81% [14]. These risks experienced by pregnant individuals using substances are further exacerbated by the experience of stigma. An individual’s perception or experience of stigma from substance use is associated with lower self-efficacy and esteem [15], prolonged treatment trajectories [16], and avoidance of treatment [17]. Fear of stigma and punitive measures resulting from substance use in pregnancy serve as barriers to prenatal care [18, 19]. Pregnant individuals using substances are more likely to delay prenatal care initiation or avoid it altogether [12, 20, 21] with one study finding this population was 2.88 times more likely to receive inadequate prenatal care [19]. Discrepancies in care received during pregnancy prevent access to treatment and care coordination that have the potential to mediate the relationship between substance use and maternal/fetal outcomes [19].

Experiences of stigma for individuals who use substances differ based on factors such as age, race, and substance type. One study found that individuals in early to mid-adulthood (26–44 years old) experienced significantly more enacted stigma (past experience) related to substance use and poorer substance use outcomes compared to younger adults (18–25 years old) [22]. However, the opposite was true for experiences of self-stigma, where younger adults in this same study reported significantly higher levels of self-stigma compared to older age groups [22]. Previous studies reveal racial/ethnic disparities, with experiences of mandated reporting being higher in people of color than in White individuals [23, 24]. Stigmatization of substances carries different weights depending on the type of substance being used [25]. Substances considered to be more socially acceptable, like marijuana, are less stigmatized than substances like opioids and amphetamines [25]. Pregnant individuals experience stigma from multiple sources including healthcare providers, family members, and self. Some studies have indicated that healthcare providers generally express negative attitudes and less willingness to treat patients with SUD [26]. Healthcare stigma separates individuals from healthcare services and treatment leading to inadequate care and poorer maternal/fetal outcomes [17, 20, 27]. Stigma from family members also impacts SUD outcomes; one study showed strong, positive associations between increased enacted stigma from family and an individual’s continued substance use indicating the negative impact that family stigma has on recovery [28]. Additionally, the social rejection experienced by individuals using substances from their family or social support system further complicates recovery [27].

Pregnancy is an important window for SUD treatment initiation, as studies show that pregnant individuals who use substances report more motivation for SUD treatment than non-pregnant individuals [29]. For pregnant individuals diagnosed with SUD, the optimal treatment regimen includes early, routine, and comprehensive prenatal care including counseling and education [10, 30]. Trauma-informed treatment for pregnant individuals using substances is important for reducing barriers to care [17]. However, substance use stigma during pregnancy presents challenges for SUD treatment and prenatal care resulting in delays or absence of prenatal care [17, 20]. Experiences of stigma vary based on several demographics of individuals who use substances, such as substance type, co-occurring mental health diagnoses, age, and race/ethnicity [22–25]. Therefore, the purpose of this article is to examine differences in experiences of self-reported experiences of enacted, anticipated, and internalized stigma among pregnant individuals with SUD by demographic, pregnancy, and SUD characteristics at a predominantly Medicaid, low-income OB-GYN Center in the Upstate of South Carolina (SC).

## Methods

### Setting

The Magdalene Clinic, established in 2016, is a collaborative care model for pregnant individuals who currently use substances or have a history of using substances. Magdalene Clinic operates within a larger, predominantly Medicaid OB-GYN center in SC and serves approximately 100 to 120 patients each year. Specialized prenatal care is integrated with addiction medicine, behavioral health, and peer support services in one co-located location. The clinic aims to incorporate principles of trauma-informed care, harm reduction, and patient-centered care to reduce patient experiences of stigma. At each visit, patients are seen by an advanced practice provider or Maternal-Fetal Medicine Specialist, depending on level of risk. Patients may also choose to see a social worker, licensed professional counselor, and peer support specialist during each visit.

### Study design and participants

This cross-sectional study, which took place between 2020 and 2024, aimed to better understand differences in stigma among pregnant individuals with SUD treated at Magdalene Clinic by demographic, pregnancy, and SUD diagnosis characteristics. Participants were eligible for the study if they were: at least 18 years of age, received prenatal care services at Magdalene Clinic, demonstrated adequate English proficiency, provided written consent to participate in the research study, and had complete Substance Use Stigma Mechanism Scale (SU-SMS) data. During one of their initial prenatal care visits Magdalene Clinic, patients were asked to participate in the study and informed consent was obtained by a member of the clinical team. If patients agreed to participate in the study, they were offered the SU-SMS survey as part of the larger Magdalene Clinic program evaluation. This study was reviewed and approved by Furman University’s IRB.

### Measures and data collection

Measures included in this study were select patient demographic, pregnancy, SUD diagnosis characteristics and self-reported substance use-related stigma. The primary outcome of interest in this study was patient experiences of substance use related stigma, which was measured using the SU-SMS survey. The SU-SMS is an 18-item self-report survey. It is comprised of three stigma domains: enacted, anticipated, and internalized stigma, and is a valid, reliable tool to evaluate substance use related stigma (α = 0.90 − 0.93) [31]. Each stigma domain is measured with six questions that use a 5-point Likert scale. Enacted stigma refers to an individual’s “personal experiences of stereotyping, prejudice, and/or discrimination from others in the past or present” (1 = Never, 5 = Very often). Anticipated stigma refers to an individual’s “expectations of stereotyping, prejudice, and/or discrimination from others in the future” (1 = Very unlikely, 5 = Very likely). Internalized stigma refers to an individual’s “endorsement and application of negative feelings and beliefs about people with SUDs to oneself” (1 = Strongly disagree, 5 = Strongly agree) [31]. The enacted and anticipated stigma domains can be used to create sub-scales of experiences of stigma with healthcare providers and family members with good reliability (α = 0.90 − 0.95) [31]. Mean scores were calculated by averaging the scores of questions within each stigma domain and sub-scale (Table 1). Stigma domains and sub-scales were scored according to the Earnshaw Lab’s scoring guide for SU-SMS [32]. A higher score on the SU-SMS indicates greater levels of stigma [31].

**Table 1:** Substance use stigma mechanism scale

*Enacted Stigma*
1. Family members have thought that I cannot be trusted^a^
2. Family members have looked down on me^a^
3. Family members have treated me differently^a^
4. Healthcare workers have not listened to my concerns^b^
5. Healthcare workers have thought that I’m pill shopping, or trying to con them into giving me prescription medications to get high or sell^b^
6. Healthcare workers have given me poor care^b^

*Anticipated Stigma*
7. Family members will think that I cannot be trusted^a^
8. Family members will look down on me^a^
9. Family members will treat me differently^a^
10. Healthcare workers will not listen to my concerns^b^
11. Healthcare workers will think that I’m pill shopping, or trying to con them into giving me prescription medications to get high or sell^b^
12. Healthcare workers will give me poor care^b^

*Internalized Stigma*
13. Having used alcohol and/or drugs makes me feel like I’m a bad person
14. I feel I’m not as good as others because I used alcohol and/or drugs
15. I feel ashamed of having used alcohol and/or drugs
16. I think less of myself because I used alcohol and/or drugs
17. Having used alcohol and/or drugs makes me feel unclean
18. Having used alcohol and/or drugs is disgusting to me

^a^Question used to assess experiences of stigma with family members

^b^Question used to assess experiences of stigma with healthcare providers

Following informed consent, the SU-SMS survey was given to the patient via paper or iPad by a member of the care team during their first or second Magdalene Clinic visit. Completed surveys were uploaded to the patient’s electronic medical record (EMR) by nursing staff and entered in REDCap, a secure, medical database that was developed by Vanderbilt University, by trained research staff [33, 34].

Patient demographic, pregnancy, and SUD diagnosis characteristics were abstracted from the patient EMR and uploaded into REDCap by a trained research staff following patient delivery. Patient demographics included age, race/ethnicity, marital status, and highest level of education. Age was defined as the patient’s age in years and was calculated using date of birth and date of first Magdalene Clinic visit. Age was grouped into less than 25, 25-29.99, 30-34.99, and 35 or greater. Race/ethnicity was grouped as Non-Hispanic Black, Hispanic/Latinx, Non-Hispanic White, and Other because of lack of diversity in the sample. Marital status and highest level of education were obtained through self-reported data on the birth certificate application during the EMR abstraction. This data was not available for all individuals because of missing birth certificate applications. Marital status was grouped by single, married, separated, or other. Education level was grouped as less than high school, high school diploma/GED, and some college/college degree. Groupings were determined based on data distribution and dichotomization. Due to small sample size, individuals with some college were combined with those having an associate’s degree or higher.

Pregnancy characteristics included gravida and trimester of prenatal care initiation. Gravida was defined as the total number of pregnancies a patient had had including the current pregnancy and pregnancies that ended before delivery. Groupings of gravida were developed based on examination of data distribution for this measure resulting in groupings of 1–3, 4–6, and 7 or more. Initiation of prenatal care was defined as the trimester in which patients attended their first prenatal visit. The patient’s recorded gestation in weeks in the EMR at their first prenatal visit was used to group patients into first (< 13.99 weeks gestation), second (14-27.99 weeks gestation), or third (> 28 weeks gestation) trimesters.

SUD diagnosis was grouped into the following categories: opioid, stimulant, alcohol, combined opioid and stimulant, and other use disorders (i.e., marijuana, benzodiazepine, hallucinogen). SUD groupings were created using evidence from prior research along with birth outcomes of the Magdalene Clinic patients by SUD diagnoses. Patients with alcohol use disorder or alcohol use disorder combined with other SUD diagnoses had the highest rate of adverse birth outcomes (i.e., low birthweight and preterm birth). Therefore, all individuals with alcohol use disorder or alcohol use disorder combined with another SUD diagnosis were placed in the alcohol SUD group. All individuals with opioid use disorders, stimulant use disorders, or combined opioid and stimulant use disorders were considered part of the opioid, stimulant, and combined opioid/stimulant SUD groups respectively. Individuals were also considered a part of these categories even if they had other co-occurring SUDs such as marijuana use disorder. The remaining individuals who were not coded in any of the previous SUD groups were included in the other SUD group.

SUD diagnoses were abstracted by trained research staff through a combination of ICD-10 code diagnoses, diagnoses made by the addiction medicine partner, and review of clinician notes. Unclear diagnoses were reviewed and finalized by reaching consensus with the research and care team.

### Statistical analysis

Descriptive statistics were used to describe all variables in the study, including demographic, pregnancy, and SUD diagnoses characteristics, and stigma domains/sub-scales. One-way analysis of variance (ANOVA) with Tukey’s post hoc tests were used to examine differences in the bivariate associations between substance use related stigma and all other study variables. Significance was defined as *p* < 0.05 in this study. Levene’s test of homogeneity of variance were run for each ANOVA. Welch’s ANOVAs with Games-Howell post hoc tests were run in each case where the Levene’s test was significant. Levene’s test indicated a violation of the homogeneity of variances assumption (*p* < 0.05) [35] for the ANOVAs examining differences between SUD type (i.e., enacted, anticipated, and healthcare stigma), age (i.e., internalized stigma), race (i.e., internalized stigma), and education level (i.e., anticipated and healthcare stigma); therefore, a Welch’s ANOVA with Games-Howell post hoc test was conducted for these variables. This study was limited to bivariate testing due to power and sample size limitations. All analyses were performed using SAS software version 9.4 (SAS Institute Inc., Cary, North Carolina) in January 2025.

## Results

### Demographics, pregnancy, and SUD diagnoses characteristics

The final study sample included 226 participants. Not all study variables were obtained for the entire sample because of missingness of data from the EMR. The largest age group was 25-29.99 years of age (33.63%) followed by 30-34.99 years of age (30.09%), less than 25 years of age (21.24%), and greater than 35 years of age (15.04%) (*n* = 226). The majority of the study sample (*n* = 226) identified as being Non-Hispanic White (86.28%) followed by Non-Hispanic Black (8.41%), Hispanic/Latinx (1.77%), and those who identified as some other race/ethnicity (3.54%). Nearly three quarters of patients were single (73.87%), with the remaining 26.13% either married, separated, or some other marital status (*n* = 222). Nearly one-third of participants (31.13%) had less than a high school diploma, while 40.09% of participants had a high school diploma/GED, and 28.77% had some college/college degree (*n* = 212). More than half had a gravida of 3 or less (53.98%). Similarly, about half initiated prenatal care during their first trimester (50.44%) (*n* = 226). The most frequent SUD diagnosis was stimulant use disorder (32.30%), followed by opioid use disorder (26.11%), combined opioid/stimulant use disorder (24.78%), some other use disorder (11.06%), and alcohol use disorder (5.75%) (Table 2).

**Table 2:** Demographic, pregnancy, and SUD diagnoses characteristics of magdalene clinic patient sample^a^

Patient characteristic	Frequency	Percentage
*Age (n = 226)*		
Less than 25	48	21.24%
25-29.99	76	33.63%
30-34.99	68	30.09%
35 or greater	34	15.04%
*Race/ethnicity (n = 226)*		
Non-Hispanic White	195	86.28%
Non-Hispanic Black	19	8.41%
Hispanic/Latinx	4	1.77%
Other Race/Ethnicity	8	3.54%
*Marital status (n = 222)*		
Single	164	73.87%
Married	32	14.41%
Separated	23	10.36%
Other	3	1.35%
*Education level (n = 212)*		
Less than high school	66	31.13%
High school diploma/GED	85	40.09%
Some college or college degree	61	28.77%
*Gravida (n = 226)*		
1–3	122	53.98%
4–6	88	38.94%
7 or more	16	7.08%
*Prenatal Care Initiation (n = 226)*		
1st Trimester	114	50.44%
2nd Trimester	87	38.50%
3rd Trimester	25	11.06%
*SUD Type (n = 226)*		
Opioid	59	26.11%
Stimulant	73	32.30%
Alcohol	13	5.75%
Opioid + Stimulant	56	24.78%
Other	25	11.06%

^a^Sample size may vary per variable due to limitations of data retrieval from the EMR

### Enacted, anticipated, and internalized stigma

The stigma domain that was the highest among Magdalene Clinic patients was internalized stigma (M = 3.13, 95% CI [2.98, 3.27]), followed by enacted stigma (M = 2.40, 95% CI [2.27, 2.53]) and anticipated stigma (M = 2.00, 95% CI [1.87, 2.13]). Experiences of stigma with family members (M = 2.49, 95% CI [2.34, 2.64]) was higher than experiences of stigma with healthcare providers (M = 1.90, 95% CI [1.78, 2.03]) (Table 3).

**Table 3:** Mean score by stigma domain and sub-scale for magdalene clinic patient sample

Stigma domain/sub-scales (*n* = 226)	Mean	95% CI
Enacted Stigma	2.40	2.27, 2.53
Anticipated Stigma	2.00	1.87, 2.13
Internalized Stigma	3.13	2.98, 3.27
Family Stigma	2.49	2.34, 2.64
Healthcare Stigma	1.90	1.78, 2.03

Enacted and anticipated stigma scores were significantly different by SUD. Individuals with opioid (M = 2.48, 95% CI [2.23, 2.73]), stimulant (M = 2.52, 95% CI [2.29, 2.74]), or combined opioid/stimulant (M = 2.52, 95% CI [2.27, 2.78]) use disorders had significantly (*p* < 0.05) higher enacted stigma scores compared to those with other substance use disorder (M = 1.73, 95% CI [1.35, 2.12]). Results for anticipated stigma were similar to that of enacted stigma. Individuals with an opioid use disorder (M = 2.14, 95% CI [1.88, 2.39]), stimulant use disorder (M = 2.07, 95% CI [1.84, 2.30]), or combined opioid/stimulant use disorder (M = 2.12, 95% CI [1.86, 2.38]) had significantly higher anticipated stigma scores compared to individuals with an alcohol use disorder (M = 1.22, 95% CI [0.67, 1.76], *p* < 0.001) or other use disorders (M = 1.61, 95% CI [1.21, 2.00], *p* < 0.05) (Table 4). There were no statistically significant differences in enacted or anticipated stigma scores by age, race/ethnicity, marital status, education level, gravida, or trimester of prenatal care initiation.

**Table 4:** One-way ANOVAs between demographic, pregnancy, and SUD characteristics and stigma domain^a^

	Enacted		Anticipated		Internalized	
Patient characteristic	Mean	95% CI^b^	Mean	95% CI^b^	Mean	95% CI^b^
*Age (n = 226)*						
Less than 25	2.25	1.96, 2.54	1.95	1.66, 2.24	3.19	2.88, 3.50
25-29.99	2.41	2.18, 2.64	1.97	1.74, 2.20	3.12	2.87, 3.37
30-34.99	2.42	2.18, 2.66	2.02	1.78, 2.27	3.03	2.77, 3.29
35 or greater	2.54	2.20, 2.88	2.09	1.74, 2.43	3.24	2.87, 3.61
*Race/Ethnicity (n = 226)*						
Non-Hispanic White	2.45	2.31, 2.59	2.04	1.90, 2.18	3.18	3.03, 3.34
Non-Hispanic Black	1.91	1.46, 2.36	1.67	1.21, 2.12	2.75	2.26, 3.24
Hispanic/Latinx	2.21	1.22, 3.19	1.00	0.01, 2.00	2.79	1.72, 3.86
Other Race/Ethnicity	2.46	1.76, 3.15	2.31	1.61, 3.02	2.79	2.04, 3.55
*Marital Status (n = 222)*						
Single	2.33	2.17, 2.48	1.92	1.77, 2.07	3.13	2.96, 3.30
Married	2.30	1.95, 2.64	2.01	1.66, 2.35	3.14	2.76, 3.52
Separated	2.76	2.35, 3.17	2.26	1.85, 2.67	3.03	2.58, 3.48
Other	2.94	1.82, 4.07	1.78	0.65, 2.91	2.33	1.09, 3.57
*Education Level (n = 212)*						
Less than high school	2.24	2.00, 2.48	1.88	1.64, 2.12	3.15	2.88, 3.41
High school diploma/GED	2.29	2.07, 2.50	1.96	1.75, 2.17	3.04	2.80, 3.27
Some college or college degree	2.60	2.35, 2.85	2.02	1.77, 2.27	3.10	2.82, 3.37
*Gravida (n = 226)*						
1–3	2.40	2.22, 2.58	1.98	1.80, 2.16	3.20	3.01, 3.39
4–6	2.42	2.21, 2.63	2.09	1.88, 2.30	3.11	2.88, 3.33
7 or more	2.23	1.73, 2.72	1.69	1.19, 2.19	2.68	2.14, 3.21
*Prenatal Care Initiation (n = 226)*						
1st Trimester	2.38	2.20, 2.57	1.91	1.72, 2.10	2.87^c^	2.68, 3.07
2nd Trimester	2.41	2.20, 2.62	2.09	1.88, 2.31	3.28	**3.06**, 3.50*
3rd Trimester	2.43	2.03, 2.82	2.11	1.71, 2.51	3.73	**3.32**, 4.15**
*SUD Type (n = 226)*						
Opioid	2.48	**2.23**, 2.73**	2.14	**1.88**, 2.39*^,^**	2.97	2.69, 3.25
Stimulant	2.52	**2.29**, 2.74**	2.07	**1.84**, 2.30*^,^**	3.27	3.02, 3.52
Alcohol	2.08	1.54, 2.61	1.22^c^	0.67, 1.76	2.99	2.39, 3.58
Opioid + Stimulant	2.52	**2.27**, 2.78**	2.12	**1.86**, 2.38*^,^**	3.18	2.89, 3.47
Other	1.73^c^	1.35, 2.12	1.61^c^	1.21, 2.00	3.03	2.60, 3.46

^a^Sample size may vary per variable due to limitations of data retrieval from the EMR

^b^Boldface indicates statistical significance (*p < 0.05, **p < 0.001)

^c^Comparison group

Internalized stigma scores were significantly different by prenatal care initiation. Individuals who started prenatal care in either their second (M = 3.28, 95% CI [3.06, 3.50], *p* < 0.05) or third trimester (M = 3.73, 95% CI [3.32, 4.15], *p* < 0.001) had significantly higher internalized stigma scores than individuals who started care in their first trimester (M = 2.87, 95% CI [2.68, 3.07]) (Table 4). There were no statistically significant differences in internalized stigma scores by age, race/ethnicity, marital status, education level, SUD, or gravida.

Enacted/anticipated stigma from family members and healthcare providers varied significantly by SUD. Individuals with stimulant use disorder (M = 2.77, 95% CI [2.51, 3.03], *p* < 0.05) reported higher levels of enacted/anticipated stigma from family members compared to individuals with some other SUD (M = 2.03, 95% CI [1.59, 2.48]). Individuals with alcohol use disorder (M = 1.35, 95% CI [0.85, 1.85], *p* < 0.001), stimulant use disorder (M = 1.82, 95% CI [1.61,2.04], *p* < 0.05), and some other SUD (M = 1.31, 95% CI [0.95, 1.67], *p* < 0.001) reported lower levels of enacted/anticipated stigma from healthcare providers compared to those with opioid use disorder (M = 2.20, 95% CI [1.97, 2.44]). Individuals with a combined opioid/stimulant use disorder (M = 2.10, 95% CI [1.85, 2.34]) or stimulant use disorder (M = 1.82, 95% CI [1.61,2.04]) reported significantly higher levels of enacted/anticipated stigma from healthcare providers compared to those with alcohol use disorder (M = 1.35, 95% CI [0.85, 1.85], *p* < 0.05) or some other SUD (M = 1.31, 95% CI [0.95, 1.67], *p* < 0.001). Additionally, those with higher educational attainment such as high school diploma/GED (M = 1.94, 95% CI [1.74, 2.14]) or some college/college degree (M = 2.02, 95% CI [1.78, 2.25]) reported significantly higher levels of enacted/anticipated stigma from healthcare providers than those with less than a high school diploma/GED (M = 1.64, 95% CI [1.42, 1.87], *p* < 0.05) (Table 5). There were no differences in enacted/anticipated stigma levels from family members or healthcare providers by age, race/ethnicity, marital status, gravida, or trimester of prenatal care initiation.

**Table 5:** One-way ANOVAs between demographic, pregnancy, and SUD characteristics and family and healthcare stigma sub-scales^a^

	Family		Healthcare	
Patient characteristic	Mean	95% CI^b^	Mean	95% CI^b^
Age (*n* = 226)				
Less than 25	2.52	2.19, 2.84	1.68	1.41, 1.95
25-29.99	2.48	2.22, 2.74	1.90	1.69, 2.12
30-34.99	2.50	2.23, 2.78	1.94	1.72, 2.17
35 or greater	2.48	2.09, 2.86	2.15	1.83, 2.47
*Race/Ethnicity (n = 226)*				
Non-Hispanic White	2.53	2.37, 2.69	1.95	1.82, 2.09
Non-Hispanic Black	2.06	1.55, 2.58	1.52	1.09, 1.95
Hispanic/Latinx	2.00	0.88, 3.12	1.21	0.27, 2.15
Other Race/Ethnicity	2.81	2.02, 3.61	1.96	1.30, 2.62
*Marital Status (n = 222)*				
Single	2.42	2.24, 2.59	1.83	1.69, 1.97
Married	2.39	1.99, 2.78	1.92	1.59, 2.24
Separated	2.91	2.44, 3.37	2.12	1.73, 2.50
Other	2.56	1.27, 3.84	2.17	1.10, 3.23
*Education Level (n = 212)*				
Less than high school	2.47	2.20, 2.75	1.64^c^	1.42, 1.87
High school diploma/GED	2.31	2.06, 2.55	1.94	**1.74**, 2.14*
Some college or college degree	2.61	2.32, 2.89	2.02	**1.78**, 2.25*
*Gravida (n = 226)*				
1–3	2.48	2.28, 2.69	1.90	1.73, 2.07
4–6	2.59	2.35, 2.83	1.92	1.72, 2.13
7 or more	2.06	1.50, 2.62	1.85	1.38, 2.33
*Prenatal Care Initiation (n = 226)*				
1st Trimester	2.43	2.22, 2.64	1.86	1.68, 2.04
2nd Trimester	2.54	2.30, 2.79	1.96	1.76, 2.16
3rd Trimester	2.61	2.16, 3.07	1.92	1.54, 2.30
*SUD Type (n = 226)*				
Opioid	2.42	2.13, 2.71	2.20^c^	1.97, 2.44
Stimulant	2.77	**2.51**, 3.03*	1.82^c^	**1.61**, 2.04*
Alcohol	1.95	1.33, 2.56	1.35	**0.85**, 1.85*^,^**
Opioid + Stimulant	2.55	2.25, 2.85	2.10^c^	1.85, 2.34
Other	2.03^c^	1.59, 2.48	1.31	**0.95**, 1.67**

^a^Sample size may vary per variable due to limitations of data retrieval from the EMR

^b^Boldface indicates statistical significance (*p < 0.05, **p < 0.001)

^c^Comparison group

## Discussion

The purpose of this study was to examine the differences in enacted, anticipated, and internalized stigma experienced by pregnant individuals experiencing SUD, who received prenatal care at Magdalene Clinic. In general, people experiencing SUD are stigmatized at high rates in society, where SUD is often viewed as a personal weakness or a moral failure [17]. Research shows that pregnant or parenting individuals are often stigmatized at higher rates due to social norms and ideals associated with motherhood [7, 17]. Similar to previous research, this study found that Magdalene Clinic patients experienced high rates of stigma related to substance use, with internalized stigma occurring more frequently compared to other types of stigma.

Internalized stigma is known to have deeply rooted impacts at the individual level, where people believe they deserve mistreatment and accept injustices that are done to them [36]. Other studies have found that internalized stigma has detrimental effects on healthcare accessibility, mental health outcomes, and overdose risk [37]. Specifically, individuals with SUD who experience internalized stigma are less likely to seek treatment for SUD and prenatal care [17]. Unsurprisingly, the results of this study showed that individuals who initiated prenatal care in the second and third trimesters had higher rates of internalized stigma compared to those who initiated care in the first trimester. These findings highlight the need for more community-based interventions to reduce internalized stigma related to substance use among individuals of childbearing age, while also supporting the need for better educating prenatal care providers on how to empathetically care for and address the specific SUD related treatment needs of these individuals [17, 38].

Interestingly, this study found that participants experienced anticipated stigma the least. Consistent with prior research, our findings reflect how enacted, anticipated, and internalized stigma are deeply interconnected, experiencing stigma in one context can heighten expectations of future discrimination, reinforcing self-blame and internalized stigma [39]. This cyclical process often operates within broader structural forces, where systemic stereotyping and criminalization of substance use create top-down effects that shape how individuals perceive themselves and are treated by others [39]. The intersection of substance use stigma with gendered and maternal stigma further compounds these experiences, particularly among pregnant or parenting individuals who use substances. Previous studies have shown that women in these contexts face moral judgment and surveillance that are not only punitive but also intertwined with societal expectations of motherhood [7, 8]. These intersecting stigmas are intensified by policy environments, such as those influenced by Nixon’s “War on Drugs”, that disproportionately criminalize substance use among women and people of color [40, 41]. Criminalization of illicit substances also leads to increased stigma for individuals who use these types of substances [42, 43], hindering access to care for fear of punishment [18, 19]. In states like South Carolina, where substance use during pregnancy can be prosecuted as child abuse [44–47], these overlapping stigmas reinforce fear, mistrust, and avoidance of care, underscoring the complex, intersectional nature of stigma experienced by this group.

Our findings indicate that participants with alcohol use disorder reported lower stigma scores compared to those using illicit substances, despite alcohol use disorder being linked to worse birth outcomes [48]. These results suggest that stigma toward illicit substances is shaped more by societal legal perceptions of substances rather than actual health risks they pose. Similar to the results of our study, previous research indicates individuals who use illicit opioids or stimulants, like heroin, fentanyl, or methamphetamines are stigmatized at higher levels by family members and healthcare providers [25, 49]. These findings indicate the need for prenatal SUD treatment that considers the entire family unit or social support system, not just the pregnant individual as the compounded effects of stigma can have detrimental impacts on the health of pregnant individuals and the generations of children that come after them [17].

Interestingly, stigma in this sample was not limited to those with traditionally higher vulnerability; participants with higher educational attainment reported more enacted/anticipated stigma from healthcare providers than those with less than a high school diploma. Higher education is generally thought to create social advantages; our findings suggest that more educated individuals may face unique stigma, possibly because society expects more of them [50, 51]. This shows that stigma affects people across all educational levels highlighting the importance of provider training that addresses the diverse experiences of pregnant individuals with SUD, rather than assuming only those with less education are vulnerable stigma’s effects [50, 51].

### Limitations

This study emphasized the compounded effects of stigma at the intersection of pregnancy and experiencing SUD; however, it was not without limitations. These results cannot be considered generalizable to all individuals experiencing pregnancy and SUD simultaneously, as other variables must be considered in targeted program and policy solutions. While not eliminating experiences of stigma, Magdalene Clinic’s collaborative, integrated care approach may have influenced perceptions of stigma in this study. Sample size is also a limitation in this study as such multivariate analyses were not appropriate due to low power. However, integrated healthcare for both SUD and pregnancy is a novel, underutilized solution, so smaller sample sizes were expected. SUD diagnosis is a complex variable to explore in research, as individuals can use multiple substances at one time, making the creation of ordinal variables difficult, which can introduce bias into the results. Lastly, the SU-SMS has several items that contribute to multiple subscales, so differences across stigma types may partly reflect item duplication.

## Conclusion

The implications of this study highlight the complex, multifaceted nature of stigma experienced by pregnant individuals with SUD, especially within healthcare settings. These findings emphasize the need for targeted interventions, like Magdalene Clinic, to reduce stigma within prenatal care settings to ensure timely, adequate healthcare access. Mental healthcare should be integrated with both SUD and pregnancy services to address the stigma-related distress that patients may be facing. The intersectionality of stigma, pregnancy, and SUD should be further examined to better understand meaningful solutions for both pregnant individuals and future generations. This study highlights the need for broader policy changes; particularly decriminalizing substance use and promoting supportive frameworks that emphasize rehabilitation and care over punishment. Policy reform could help alleviate some of the societal stigmas associated with both pregnancy and SUD. Overall, the study calls for a more empathetic, inclusive approach in healthcare, with a focus on reducing stigma at both the individual and systemic levels. This would improve care for pregnant individuals with SUD and help mitigate the broader health consequences that can arise from stigmatization.

## Data Availability

The datasets used and/or analyzed during the current study are available from the corresponding author on reasonable request.

## Abbreviations

SUD: Substance use disorder
SC: South Carolina
SU-SMS: Substance use stigma mechanism scale
EMR: Electronic medical record
ANOVA: Analysis of variance
